# Robust Findings From 25 Years of PTSD Genetics Research

**DOI:** 10.1007/s11920-018-0980-1

**Published:** 2018-10-23

**Authors:** Laramie E. Duncan, Bryna N. Cooper, Hanyang Shen

**Affiliations:** 1 Department of Psychiatry and Behavioral Sciences, Stanford University, 401 Quarry Road, Room 3320, Stanford, CA 94305 USA; 2 PGSP-Stanford Psy.D Consortium, Palo Alto, CA USA

**Keywords:** Posttraumatic stress disorder, PTSD, Genetics, Genome-wide association study, GWAS, Polygenic

## Abstract

**Purpose of Review:**

The purpose of this review is to contextualize findings from the first 25 years of PTSD genetics research, focusing on the most robust findings and interpreting results in light of principles that have emerged from modern genetics studies.

**Recent Findings:**

Genome-wide association studies (GWAS) encompassing tens of thousands of participants enabled the first molecular genetic heritability and genetic correlation estimates for PTSD in 2017. In 2018, highly promising loci for PTSD were reported, including variants in and near the *CAMKV*, *KANSL1*, and *TCF4* genes.

**Summary:**

Twin studies from 25 years ago established that PTSD is genetically influenced and foreshadowed the molecular genetic findings of today. Discoveries that were impossible with smaller studies have been achieved via collaborative/team-science efforts. Most promisingly, individual genomic loci offer entirely novel clues about PTSD etiology, providing the raw material for transformative discoveries, and the future of PTSD research is bright.

## Introduction

### Background

Posttraumatic stress disorder (PTSD) is categorized by the *fifth edition of the Diagnostic and Statistical Manual of Mental Disorders* as a Trauma- and Stressor-Related Disorder. It occurs in a subset of people exposed to a traumatic event, leading to a subsequent response pattern including persistent re-experiencing of the trauma, avoidance of trauma-related stimuli, negative thoughts and feelings, and trauma-related arousal and reactivity [1]. Exposure to potentially traumatic events is common, with 70.4% of individuals worldwide reporting traumatic experience at least once in their lifetime. Of these individuals, a small but substantial portion develop PTSD. The lifetime prevalence of PTSD worldwide is 4% [2, 3]. As is the case in many developed countries, the USA has a slightly elevated lifetime prevalence of PTSD compared to the global rates. In the USA, 83% of citizens report at least one trauma exposure in their lifetime, and of those individuals, lifetime prevalence for PTSD is 6.8% [2, 4, 5].

Trauma exposure, as well as PTSD risk conditional on exposure, occurs unevenly across trauma types, environmental factors, and individual differences. The type of trauma experienced, for one, greatly impacts the severity and duration of PTSD symptoms. Kessler and colleagues [2] found that 4% of individuals develop PTSD when all trauma types are analyzed together. When exposed to physical or sexual abuse by an intimate partner, however, risk is higher (approximately 11%). The risk of developing PTSD is highest among rape victims (19%), and much lower for many other trauma categories including accidents (2%), natural disasters (0.3%), and traumas that were witnessed but not directly experienced (2.4%). When trauma type is held constant, age is a significant predictor for developing PTSD, wherein risk is particularly high among children, adolescents, and older adults [2, 6]. Gender is also largely predictive of PTSD risk; in that lifetime, prevalence for women is 8.5% compared to 3.4% for men [2, 7, 8]. This is partially due to the fact that women disproportionately experience the types of trauma that are most likely to precipitate PTSD (i.e., physical and sexual abuse by an intimate partner and rape). Thus, societal patterns of violence partially explain the increased rate of PTSD among women compared to men. Other associated factors that appear to be implicated in increasing PTSD risk include lower socioeconomic status, fewer years of education, lack of social support, and lower social status [6, 8, 9].

Given that PTSD is primarily understood in terms of trauma exposure and relevant environmental factors, genetic studies may seem unnecessary. However, genetic studies afford unique opportunities to understand individual differences in risk and resilience to PTSD. Further, genetic approaches are capable of revealing underlying biological mechanisms contributing to the development, maintenance, and resolution of PTSD. Further, clues about the molecular etiology of PTSD, revealed through genetic studies, may facilitate the development of novel drugs that improve treatment of PTSD. Thus, genetic research on PTSD may ultimately enable prevention, early detection, and improved treatments for sufferers of PTSD.

### Scope of Review

The scope of this review is broad in terms of the years covered, but narrow in its focus on the most robust findings available. We review all genetic studies of PTSD to date,^1^ a time-frame which corresponds to the first 25 years of PTSD genetics research. The first twin study of PTSD was published in 1993 [10], and critical milestones in the arc of molecular genetic research about PTSD were published in 2017 [11••] and 2018 [12••, 13••] (namely, genome-wide association studies (GWAS) of tens of thousands of participants were finally completed). The vantage point made possible by recent genetics findings [14••, 15, 16•, 17•] allows us to see which findings withstood the test of time, or are likely to do so, as compared to those that have not withstood the test of time (or are unlikely to do so). In brief, this means that we focus on twin studies of PTSD and large-scale genetic studies of PTSD, while omitting candidate gene studies and “small” GWAS (i.e., those with only thousands of participants, or less). The last decade has witnessed unprecedented advances in the field of genetics—in both technology and fundamental principles that have emerged—and we use this post- genomics revolution perspective [14••, 15, 18, 19, 20•] to make sense of the first 25 years of PTSD genetics research.

### Background Information

In the service of orienting readers to this review, it is worth noting that many commonly held expectations about genetic influences on psychiatric disorders have been substantively revised in the last few years, based on findings from large-scale molecular genetic studies [14••, 15, 16•, 17•]. In reading this review, it is helpful to be aware of certain terms (e.g., “complex genetic phenotypes” and “polygenicity”). As well, we note major shifts in expectations (i.e., about polygenicity, effect sizes, and the genes relevant to psychiatric disorders) and advances in research methodology (e.g., the necessity of very large sample sizes).

Complex genetic phenotypes are those phenotypes that are influenced by both environmental factors and many genetic variations. Notable examples include height, depression, cardiovascular disease, personality traits, and all psychiatric disorders. Though scientists do not fully understand the causes of the highly polygenic genetic architecture of complex genetic traits, it is clear that these phenotypes are fundamentally similar to one another in the following ways. First, geneticists no longer expect a small number of “genes for” complex genetic phenotypes like PTSD, rather, available evidence suggests that *thousands* of genetic variants likely impact risk for PTSD [16•, 23, 24]. Second, we expect that many PTSD risk loci will be located in introns (i.e., the non-coding portions of genes) or outside of genes altogether, in the genomic regions that were previously referred to as “junk DNA” (i.e., intergenic regions, the functions of which are still generally poorly understood) [25]. Finally, we expect that all PTSD risk variants will have small effects on population risk for PTSD. Specifically, most genetic risk variants will have odds ratios of 1.05 and lower (per risk allele, for common variants), and the largest effect sizes for common variants will likely have odds ratios < 1.30.

Research practices have changed dramatically in recognition of the challenges posed by small effect sizes on complex genetic phenotypes. In other words, methods have been adapted in light of constraints imposed by nature. Most notably, researchers have formed consortia and have created national biobanks in order to achieve the necessary sample sizes of tens of thousands, to millions of people. For example, a forthcoming genetic study of human height will include millions of individuals, and the largest study of depression, just published, included approximately 460,000 individuals [26••]. The exciting news for PTSD research is that sufficient sample sizes have just been achieved, and these efforts are making foundational discoveries about PTSD genetics possible.

## Twin Studies of PTSD

Historically, twin studies provided the first window into the genetic basis of PTSD. Twin studies leverage an experiment afforded by nature: monozygotic (identical) and dizygotic (fraternal) twins share the same life time-course, starting with shared time in the womb. In contrast, monozygotic twins share 100% of inherited nuclear DNA, whereas dizygotic twins share only 50%, on average. Thus, environmental similarity is assumed to be equivalent for monozygotic and dizygotic twins, whereas genetic similarity is twice as high in monozygotic twins compared to dizygotic twins. Using biometrical modeling, scientists can obtain estimates of the relative contributions of genetic and environmental influences, and heritability is the term used to describe phenotypic variance attributable to genetic variance.

The twin study design is simple, and yet twin studies retain an advantage over even the most sophisticated molecular genetic approaches available today. Specifically, twin studies capture all inherited genetic effects, including those attributable to common and rare genetic variations, as well as complex genetic variations (which can be of any frequency). In contrast, GWASs primarily assess common genetic variation (i.e., variants with minor allele frequencies greater than approximately 1%), and measurement of many variants is imperfect. Thus, the best available estimates of heritability are obtained using twin studies, rather than molecular genetic studies. Molecular genetic studies play a necessary role in the validation of twin study results and, most importantly, are needed for the discovery of specific genetic risk variants.^2^

**Fig. 1:**
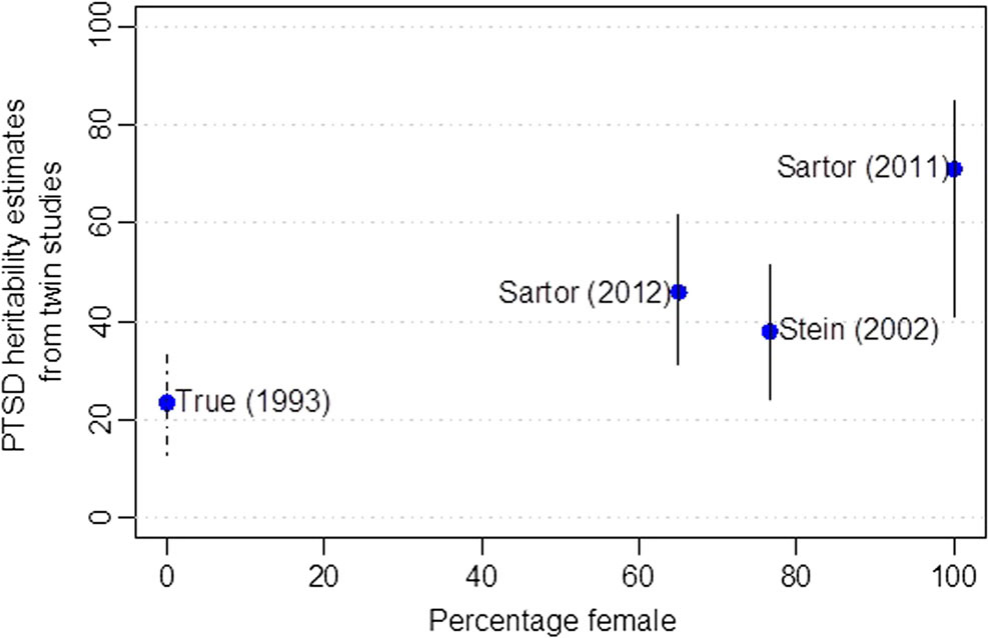
PTSD twin study heritability estimates (*h*^2^_twin_) from independent samples, plotted against percentage of female participants. Blue dots represent point estimates for *h*^2^_twin_. Solid vertical lines denote the 95% confidence interval for *h*^2^_twin_ estimates. The dotted vertical line (for True et al.) denotes the range of *h*^2^_twin_ estimates reported for PTSD symptoms (95% confidence interval not given in original report)

Twin study heritability estimates (*h*^2^_twin_) and sample characteristics for four major twin studies of PTSD [10, 27–29] are provided in Table 1. The first study, by True and colleagues [10], focused on the Vietnam Era Twin Registry, and five additional studies [30–34] have also examined participants from the Vietnam Era Twin Registry (i.e., samples are overlapping for these studies). The studies in Table 1 examined non-overlapping samples, and all found evidence of genetic effects on PTSD. Point estimates for heritability ranged from a low of 23.5% (average of symptom *h*^2^_twin_ estimates from True et al.) to a high of 71% for Sartor and colleagues’ investigation of PTSD in women. Further research is needed to determine the reasons for differences in heritability estimates across studies, and sampling variability is undoubtedly one of the explanations. In addition, preliminary evidence is consistent with differences in heritability by sex/gender, with higher heritability estimates among females. Figure 1 provides point estimates of *h*^2^_twin_ from these four studies, plotted against the percentage of female participants in each study. The trend for higher twin heritability estimates (*h*^2^_twin_) among females, shown in Fig. 1, is also consistent with molecular genetic heritability estimates (*h*^2^_SNP_) as described below. Nevertheless, more research is needed to determine if real differences in PTSD heritability exist across subpopulations, and the major take-home message from all available twin studies is that PTSD is partially genetically influenced. In other words, genetic variation underlies individual differences in risk/resilience to PTSD.

**Table 1:** Major twin studies of PTSD, from independent samples

Author	Year	Population	Twin pairs	Female (%)	PTSD heritability	95% PTSD *h*^2^_twin_ CI
True et al.	1993	US Vietnam Era Twin Registry	4042	0	23.5%	13–34%*
Stein et al.	2002	Vancouver area, Canada	406	76.6	38%	24–52%
Sartor et al.	2011	Missouri, USA	1772	100	71%	41–85%
Sartor et al.	2012	Australian national sample	766	65	46%	31–62%

*PTSD*, posttraumatic stress disorder; *CI*, confidence interval; *US*, United States; *h*^*2*^_*twin*_, twin study-based heritability estimate. *Not a confidence interval; rather, this is the range of heritability values for PTSD symptoms in this study, because confidence intervals were not given in True et al.

Twin studies revealed genetic effects on PTSD risk following trauma exposure, and they also revealed genetic effects on trauma exposure itself [10, 27–29], meaning that trauma exposure is partially heritable. In general, studies have revealed higher heritability for interpersonal trauma types than for trauma related to natural disasters and accidents [27–29]. The latter are presumably more random and less influenced by individual characteristics, and this could explain lower heritability estimates for natural-disaster and accident-related trauma, as compared to interpersonal trauma types.

Findings about genetic effects on trauma exposure are consistent with a robust body of literature about modest genetic influences on environmental exposures such as stressful life events [35, 36]. The literature about genetic effects on “environmental” exposures suggests that some risk loci will influence trauma exposure, others will influence PTSD risk following trauma, and still others will influence both trauma exposure and subsequent risk for developing PTSD. Thus, a complete understanding of genetic effects on PTSD will ultimately include knowledge about specific genetic effects on trauma exposure and specific genetic effects on the subsequent development of PTSD. Underlying mechanisms are likely to be partially shared and partially distinct.

## Molecular Genetic Studies of PTSD

### Genome-Wide Association Study Methodology and Overview

The human genome contains millions of loci that are commonly polymorphic across people (e.g., single nucleotide polymorphisms, SNPs) and modern genetic methods (e.g., GWAS) are capable of detecting associations between these genetic variants and complex genetic phenotypes, including PTSD. GWASs have been tremendously successful in recent years, identifying thousands of genetic variants associated with complex genetic phenotypes [14••, 15]. This includes over 200 risk loci for psychiatric disorders discovered already [26••, 37–40]. Notably, most of these discoveries were impossible with modest GWAS that included only thousands of individuals. In addition, just as petroleum engineers can make accurate predictions about how much oil is left in the ground in a particular area (using data and statistical models), statistical geneticists can now predict the likely number of genetic risk variants for a given phenotype that can ultimately be discovered [16•, 37, 41]. Concrete evidence supporting such predictions is already available for phenotypes including height [42] and schizophrenia [37].

Overall, GWAS results have revealed certain points and principles about genetic effects on psychiatric disorders, including the expectation that phenotypes like PTSD are highly polygenic and that there are strict bounds on variant effect sizes (as described above). GWAS results have also revealed complexity in understanding robust GWAS results, even after loci are identified. For example, it is difficult to find the true risk variant(s) within a given GWAS locus. Further, determining the functional effect of a true risk variant is also difficult. These points are described further below, as they pertain to PTSD.

### Candidate Gene Study Methodology and Overview

With the current availability of affordable, high-coverage genotyping and analysis methods (e.g., GWAS), candidate gene studies are no longer necessary. Candidate gene studies measure only small numbers of genetic variations (typically one to ten polymorphisms, out of the many millions of common polymorphisms that exist). In contrast, each GWAS typically encompasses *all* candidate polymorphisms, as well as thousands of variations in and around each candidate gene, and also millions of other genetic variants throughout the genome. Thus, the genomic coverage afforded by GWAS makes candidate gene studies obsolete. A second reason why candidate gene studies are no longer recommended is that GWAS data affords far superior analytical procedures. Simply put, it is impossible to correct for known confounders to genetic studies (i.e., population stratification and subtle relatedness) using candidate gene data alone. Finally, the other reason why geneticists routinely disregard candidate gene findings is that the replication record of candidate gene studies has been notoriously poor. Given that GWAS results have shown that candidate gene hypotheses were typically wrong in two major ways (i.e., they specified the wrong polymorphisms and also explicitly or implicitly hypothesized effect sizes that were too large), we now have a sensible explanation for the poor replication record of candidate gene findings, namely, that nearly all results were false positives [18, 19, 20•, 43–46]. Exceptions to this poor replication record exist for substance-related phenotypes, in which some of the correct genes (though perhaps not the correct polymorphisms) have been previously hypothesized (e.g., ADH1B associations to alcohol phenotypes [47–50]). Given these points, we do not review candidate gene findings for PTSD. Fortunately however, the first robust findings from GWAS of PTSD are just emerging, and so we review available and forthcoming findings below.

### Robust Molecular Genetic Findings for PTSD Are Emerging

#### Million Veterans Program

The Million Veterans Program [51] (MVP) biobank is one of the world’s leading repositories of genetic and phenotypic information, and is an unprecedented resource for the study of PTSD. A conference abstract on MVP GWAS of PTSD re-experience symptoms has been published [12••] and additional results about other PTSD phenotypes measured within MVP will be available in future publications. Regarding PTSD re-experiencing symptoms, MVP researchers examined a sample of 146,660 European-ancestry participants and 19,983 African-ancestry participants. This dataset afforded the discovery of eight loci at the level of genome-wide significance (i.e., *p* < 5 × 10^−8^). These loci include a chromosome 3 locus with top variant rs2777888 (*p* = 2.1 × 10^−11^). This variant is located in an intron of the *CAMKV* gene (CaM kinase like vesicle associated), which is highly expressed in the brain. An extended locus on chromosome 17 was also identified, with lead SNP rs2532252 (*p* = 4.5 × 10^−10^) closest to the *KANSL1* gene (KAT8 regulatory NSL complex subunit 1). This locus also encompasses the *CRHR1* gene (corticotropin releasing hormone receptor 1), a previous candidate gene for PTSD. This means that CRHR1 may be associated with PTSD, but further research is needed to assess this possibility given the large number of genes and regulatory regions in this broad locus (see notes about fine-mapping below). A third locus on chromosome 18 is located in a locus previously associated with schizophrenia, in the *TCF4* gene (transcription factor 4; top PTSD SNP rs2123392, *p* = 5.4 × 10^−11^). The discovery of these loci [12••] is a tremendous step forward for PTSD genetics. The forthcoming full manuscript, as well as further genetic studies of PTSD phenotypes from MVP, will provide motivating results for the field. The major limitation of the MVP studies is the exclusive focus on military samples. The Psychiatric Genomics Consortium studies (below) include both military and civilian samples, from a wide variety of contexts relevant to PTSD.

#### PTSD Group of the Psychiatric Genomics Consortium (PGC-PTSD) and the UK Biobank

As has been the case for much of complex trait genetics research, the formation of international consortia focused on the genetics of PTSD has been a critical step in for discovery because far larger sample sizes can be achieved through sharing of data. In psychiatry, the largest genetics consortium is the Psychiatric Genomics Consortium, PGC [41]. The PGC made possible the identification of over 100 risk loci for schizophrenia, as reported in 2014 [37], and more loci have consistently been identified as data aggregation within the PGC schizophrenia group has continued. The PGC-PTSD group [21, 22] has been employing the same strategy used by other successful PGC groups, and the first empirical paper for the group had a sample size of 20,070 [11••], from the combined analysis of 11 previous GWAS of PTSD [52–56]. This study was notable because it was the first to be adequately powered to estimate *h*^2^_SNP_ for PTSD. Intriguingly, the *h*^2^_SNP_ estimate for females (29%) was higher than the *h*^2^_SNP_ estimate for males (7%), consistent with twin study PTSD heritability estimates (i.e., *h*^2^_twin_FEMALE_ estimates are higher than *h*^2^_twin_MALE_ estimates), as described above. Analyses from the PGC-PTSD group also revealed shared genetic effects between PTSD and schizophrenia, bipolar disorder, and depression [11••, 13••].

The second wave of data analysis from the PGC-PTSD group (abstract currently available [13••], and manuscript forthcoming) replicated and extended the findings from the first PGC-PTSD paper [11••] and also identified potential specific risk loci and genes for PTSD. At the time of writing of this review, the following information about top loci is available from the published abstract [13••]. Stratified analyses revealed two loci (on chromosomes 6 and 13) that exceeded genome-wide significance in the European ancestry analyses (6q25, *p* = 3.1 × 10^−9^ and 13q32, *p* = 2.7 × 10^−8^). In the African ancestry analyses, a separate locus on chromosome 13 exceeded genome-wide significance (13q.21, *p* = 3.8 × 10^−8^). Further, polygenic analyses make it clear that the identification of many more loci will occur once adequate power is achieved. Thus, sample collection is continuing within the PGC-PTSD group. Like those loci identified by in the Million Veterans Program, the next step for the PGC-PTSD loci is “fine-mapping.” Fine-mapping [57•] refers to various analytical and biological procedures used to refine the signal within a particular locus, ideally to the resolution of individual variants causally associated with disease.

In closing this section about GWAS results, we note that the methodological advantages of GWAS (described above) do not imply that GWAS results should be accepted indiscriminately. Rather, consumers of the GWAS literature should be aware of certain guidelines in the evaluation of GWAS results. Above all, sample size has proven to be the best indicator of how many loci will be discovered and how robust findings will prove to be upon investigation in novel samples. Thus, for a given phenotype such as PTSD, a good rule of thumb is that the largest GWAS (i.e., largest N) will likely provide the best information about molecular genetic influences on PTSD. Another indicator of power in GWAS is the presence of a significant SNP-heritability estimate (*h*^2^_SNP_).

In general, smaller GWASs can be used to conduct polygenic analyses of heritability and genetic correlations, than are necessary for the identification of individual risk loci [23, 58]. Sample sizes of many tens of thousands of participants have been necessary for risk locus discovery [26••, 37], whereas methods like GCTA [23] can be used to estimate *h*^2^_SNP_ using just thousands of samples. For these reasons, we chose to focus on the MVP and PGC-PTSD GWAS results instead of smaller GWAS studies, which were not even adequately powered for heritability analyses (and by extension, it is less likely that they were adequately powered to detect individual loci). At the same time, it is important to recognize that the progress made by GWAS consortia in recent years would not have been possible without the considerable efforts involved in the conduct of each individual GWAS study. The individual GWAS that made recent consortium findings possible are provided in the reference list [52–56, 59–66] and described in detail in the PGC papers.

### Future Directions in Molecular Genetic Studies of PTSD and Related Analyses

Given that large-scale GWAS offers a proven strategy for success in the identification of risk loci for complex genetic phenotypes, the future steps for PTSD genetics are relatively clear. First, many more loci can be identified as current efforts to increase sample size continue. Based on evidence from other phenotypes, it is likely that there will ultimately be thousands of loci associated with PTSD. If we are to realize the full potential of GWAS, which is the identification of entirely novel clues about PTSD etiology, then it makes sense to identify more robust risk loci for PTSD. Next, each locus needs to be investigated using fine-mapping approaches in order to identify the causal variant(s) within each locus. In tandem, genetic analyses that use GWAS data can also identify the specific cell types relevant to PTSD [67]. Further, the strength and direction of genetic relationships between PTSD and other psychiatric and medical disorders can be discovered through genetic correlation analyses [23, 68], as has been successfully achieved for schizophrenia and many other phenotypes [67, 69–71].

In addition to the focus of this review—genetic variations—there are important related areas of inquiry, such as gene expression (i.e., transcriptomic) and epigenetic (including methylation) studies. It is important to keep in mind that the same challenges of scope apply to these fields, as they do to genetic association studies. In other words, there is a good chance that very large studies will be necessary to discover robust relationships between gene expression, methylation, and PTSD. Therefore researchers should interpret results from small studies, and those that are not widely replicated, cautiously. Indeed, work within the PGC-PTSD group (unpublished) shows that genome-wide significant methylation results from individual studies are oftentimes not consistent across studies. For this reason, they developed systematic quality control procedures for the analysis of epigenome-wide studies (EWAS) [72], and empirical results are forthcoming. An even greater consideration for gene expression, methylation, and other epigenetic studies is that results are highly variable across cell types and tissues [73–76]. Thus, results in accessible tissues like blood may only be partially correlated (if at all) with results in relevant brain cell types. Nevertheless, preliminary results are available, for example, gene expression in blood has been examined in a meta-analysis of 540 individuals, combined from multiple individual studies [77]. Finally, additional efforts are underway to quantify molecular genetic effects on trauma exposure (Dalvie et al., in prep); to assess diverse genetic correlations with PTSD (Ratanatharathorn et al., in prep); to understand relationships between genetic variation, brain imaging phenotypes, and PTSD (imaging data only has been published [78]); and to understand relationships among PTSD, genetics, sex, and gender [11••, 79]. As these efforts continue, and in particular as adequately powered studies are created, we can expect important discoveries about genetic, transcriptomic, and epigenetic influences on PTSD.

## Conclusions

It is an incredibly exciting time for PTSD genetics research because well-powered GWAS are finally delivering robust molecular genetic results for PTSD [11••, 12••, 13••, 80]. Twin studies from as long as 25 years ago portended such discoveries [10, 27–34], but genetic technology and sample sizes were inadequate—until now—for the discovery of specific risk variants for PTSD. Plainly stated, candidate gene studies and small GWASs were inadequate for the detection of most (if not all) true PTSD risk variants. We know this (in 2018) because we have the benefit of hindsight informed by the molecular genetic revolution, which was built upon dramatic advances in genotyping technology and the combined efforts of scientists that worked together to assemble adequate sample sizes (with tens of thousands of participants) via international consortia like the Psychiatric Genomics Consortium [41] (PGC), and through national efforts like the Million Veterans Project [54] and the UK Biobank [81]. These deep-coverage genotyping methods and massive sample sizes have yielded approximately ten PTSD risk loci to date [12••, 13••, 80], as well as the first molecular genetic heritability and genetic correlation estimates [11••, 80, 82].

Large-scale genetic studies published in the last year have also revealed that PTSD is fundamentally similar to other complex genetic phenotypes like schizophrenia, depression, and human height, in that PTSD is a highly polygenic phenotype that is likely influenced by thousands of loci across the genome, many or all of which fall in portions of the genome that were not historically hypothesized as being most relevant. Interestingly, some researchers view the discovery of PTSD loci in “unexpected” places as an unfortunate outcome; however, this is arguably the best possible outcome. The greatest strength of GWAS is in the method’s ability to deliver *novel* clues about etiology, based on a systematic scan of the genome. This tantalizing promise is exactly what PTSD GWAS have delivered. In revealing novel loci for PTSD, recent and emerging large-scale GWASs of PTSD have set the stage for a new era of PTSD research. To make an analogy, it is as if we had only a handful of paper maps to guide us before 2017, and then suddenly we were given Google Earth. With Google Earth, as with GWAS, we can see globally important features and patterns. With paper maps and candidate gene studies, our perspective is quite limited. These advances notwithstanding, it is clear that GWAS is only the first step. The broader field of medical genetics research suggests that translation of GWAS loci into risk variants, and then mechanistic discoveries, and finally into improved treatments will take many years and likely decades, but the first critical step has been achieved, and there is every reason to expect dozens if not hundreds more risk loci for PTSD in the coming years. In sum, it has indeed been a successful 25-year period from establishment of PTSD heritability using twin studies in 1993 [10] to the first robust molecular genetic findings in 2017 [11••] and 2018 [12••, 13••]. The field has much to be proud of in these collective accomplishments, and the future of PTSD research, informed by genomics, looks bright.

## Footnotes

1 Specifically, we review studies linking *genetic variations* with PTSD. We do not review gene expression studies nor do we review epigenetic studies, including studies of methylation.

2 Full-genome sequencing studies can theoretically provide the best heritability estimates because they assess common and rare genetic variations, but such studies are still cost prohibitive, and there are still regions of the genome that are intractable to current sequencing methods.
